# Non-Surgical Management of Scrotal Extramammary Paget Disease: A Case Report of a Cutaneous Malignancy Treated with Depth-Guided Superficial Radiation Therapy

**DOI:** 10.3390/reports9020163

**Published:** 2026-05-21

**Authors:** Douglas Jaxon Vadner, Sidney Smith

**Affiliations:** 1 Chicago Medical School, Rosalind Franklin University of Medicine and Science, North Chicago, IL 60064, USA; 2 Georgia Skin and Cancer Clinic, Savannah, GA 30415, USA

**Keywords:** extramammary paget disease, superficial radiation therapy, scrotum, CK7, cutaneous oncology, adnexal involvement

## Abstract

**Background and Clinical Significance:** Extramammary Paget disease (EMPD) is a rare cutaneous adenocarcinoma that frequently involves apocrine-rich regions and may extend beyond clinically apparent margins through adnexal structures. Surgical excision remains the standard of care; however, management can be challenging in elderly patients and in anatomically sensitive areas such as the scrotum, where morbidity and functional impairment are significant concerns. Despite increasing use of radiation-based therapies, optimal superficial radiation therapy (SRT) parameters, particularly with respect to depth of penetration, remain poorly standardized. **Case Presentation:** An 88-year-old male with a history of melanoma, non-melanoma skin cancer, and remote prostate cancer presented with biopsy-proven EMPD involving the scrotum and perineum. Imaging demonstrated no evidence of underlying or metastatic malignancy. Given lesion size (9 × 4 cm), anatomic location, and patient preference to avoid surgery, SRT was selected. The patient underwent treatment with 70 kV energy, delivering a total dose of 5440 cGy in 17 fractions (320 cGy per fraction) administered twice weekly. Energy selection was guided by the known propensity of EMPD for adnexal extension, with the aim of improving treatment coverage of potential subclinical disease. **Conclusions:** This case highlights the importance of incorporating tumor depth and adnexal involvement into treatment planning for EMPD. Depth-guided SRT may represent a viable non-surgical management strategy in carefully selected patients, particularly when surgical morbidity is a concern. These findings support a more individualized, mechanism-based approach to optimizing radiation therapy in cutaneous malignancies.

## 1. Introduction and Clinical Significance

Extramammary Paget disease (EMPD) is a rare intraepithelial adenocarcinoma that most commonly arises in apocrine-rich regions, including the groin, axilla, vulva, perineum, scrotum, and penis [1,2]. Clinically, EMPD presents as erythematous, well-demarcated plaques that may become erosive, eczematous, scaly, or ulcerated over time. Patients often report chronic pruritus, burning, pain, or oozing, and diagnosis is frequently delayed due to its resemblance to benign dermatologic conditions such as dermatitis, fungal infections, or psoriasis [1,2], with misdiagnosis rates reported as high as 93.75% in some series [3]. Lesions may be multifocal and extend beyond clinically apparent margins, including along adnexal structures such as hair follicles, contributing to challenges in complete eradication [4].

Histopathologically, EMPD is characterized by Paget cells, large, pale, vacuolated cells with prominent nuclei distributed singly or in clusters within the epidermis [2]. Immunohistochemical staining is typically positive for mucin, cytokeratin 7 (CK7), epithelial membrane antigen (EMA), and carcinoembryonic antigen (CEA) [2,5]. CK20 positivity raises suspicion for secondary EMPD associated with an underlying gastrointestinal or urothelial malignancy and should prompt further evaluation [5]. Primary EMPD arises de novo within the epidermis or its adnexal structures, whereas secondary EMPD represents epidermotropic spread from an underlying visceral malignancy, most commonly of urothelial, colorectal, or prostatic origin [5,6].

Although EMPD is typically an indolent and intraepidermal malignancy, it retains the potential for dermal invasion and progression to adenocarcinoma [2,6]. In a cohort of vulvar EMPD, invasive disease was observed in a minority of cases, with prior series reporting adenocarcinoma in approximately 4% of patients, underscoring the importance of accurate diagnosis and appropriate management [4].

Surgical excision, including Mohs micrographic surgery or wide local excision, remains the standard of care; however, recurrence rates remain significant, particularly in anatomically complex regions [1,4]. In elderly patients or those with comorbidities, surgical management may be associated with substantial morbidity, functional impairment, and delayed wound healing.

As a result, non-surgical modalities, including topical therapies such as imiquimod, laser-based approaches, and radiation therapy, have been explored as alternative treatment strategies, particularly in patients who are poor surgical candidates or decline operative intervention [1,2].

Despite increasing utilization of radiation-based approaches, there remains limited standardization regarding optimal superficial radiation therapy (SRT) parameters, particularly with respect to energy selection and depth of penetration [1,3]. Given the known propensity of EMPD to extend along adnexal structures beyond the superficial epidermis, failure to account for subclinical depth may contribute to incomplete treatment and recurrence [7].

Here, we present a case of scrotal EMPD treated with SRT using a depth-informed approach to energy selection, highlighting a practical framework for optimizing non-surgical management in select patients.

## 2. Case Presentation

**Figure 1:**
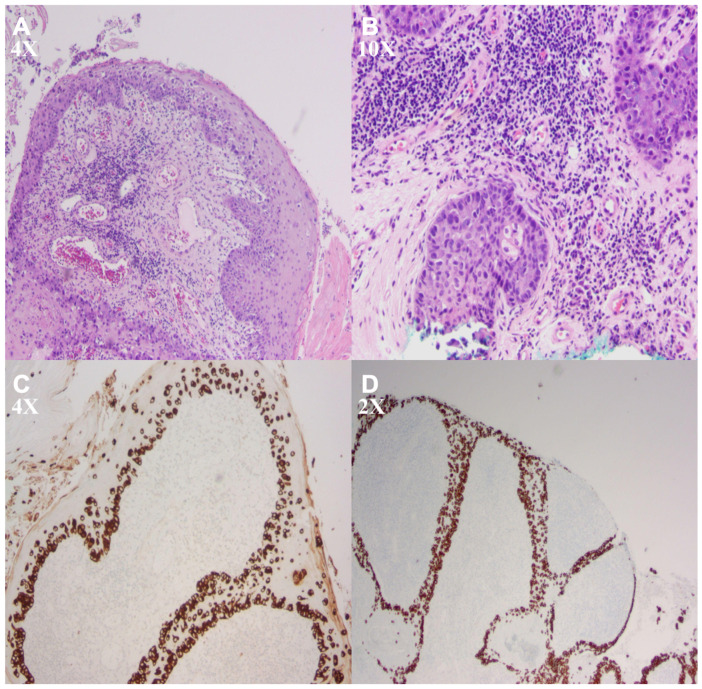
Histopathologic features of extramammary Paget disease. (**A**) Low-power H&E demonstrates intraepidermal involvement with extension along adnexal structures. (**B**) Higher magnification shows large atypical cells with abundant pale cytoplasm and prominent nuclei, consistent with Paget cells. (**C**) CK7 immunostaining highlights a pagetoid intraepidermal distribution of atypical cells. (**D**) CAM5.2 immunostaining confirms epithelial differentiation of the neoplastic cells.

An 88-year-old male with a history of melanoma, non-melanoma skin cancer, and remote prostate cancer approximately 20 years prior treated with brachytherapy presented for evaluation of a chronic scrotal and perineal lesion. Clinical differential diagnoses included chronic eczematous dermatitis, candidal intertrigo, psoriasis, contact dermatitis, and squamous cell carcinoma in situ. Histopathologic evaluation demonstrated a pagetoid intraepidermal proliferation of large, pale-staining cells. Immunohistochemical staining demonstrated strong CK7 and CAM5.2 positivity in conjunction with characteristic pagetoid histomorphology, supporting the diagnosis of extramammary Paget disease (Figure 1).

Given the association between secondary extramammary Paget disease and underlying malignancy, further evaluation was performed. Positron emission tomography-computed tomography (PET-CT) demonstrated no evidence of metabolically active metastatic disease outside of nonspecific thoracic spine uptake, with no suspicious visceral lesions or lymphadenopathy identified. Additional findings included expected post-treatment brachytherapy seeds within the prostate, along with bladder wall thickening without convincing metabolic activity, consistent with prior treatment history rather than a new primary malignancy.

The lesion measured approximately 9 × 4 cm and involved the scrotum and perineum, representing an anatomically sensitive region where surgical excision would pose a risk of functional impairment, delayed healing, poor cosmetic outcomes, and significant morbidity.

After multidisciplinary discussion, surgical excision, including Mohs micrographic surgery, was considered; however, the patient declined operative management due to concerns regarding morbidity, recovery, and functional outcomes. Alternative options, including topical therapy, were deemed less appropriate given the lesion size and suspected depth of involvement. Given the patient’s advanced age, comorbidities, and preference to avoid surgical intervention, a shared decision was made to proceed with superficial radiation therapy.

The patient underwent superficial radiation therapy using the Axxent system, delivered at 70 kV with a total prescribed dose of 5440 cGy in 17 fractions (320 cGy per fraction) administered twice weekly. Radiation field design was based on clinical examination and multidisciplinary treatment planning, without mapping biopsy. Because the lesion extended beyond the dimensions of a single treatment field, two adjacent treatment fields targeting the scrotum (6 × 6 cm) and perineum (5 × 5 cm) were utilized, incorporating approximately 1 cm treatment margins to ensure adequate coverage of the clinically involved region. Treatment planning incorporated a 10 cm applicator at a source-to-skin distance of 25 cm, with lead shielding used to protect surrounding normal tissues. Treatments were administered from 7 October 2025 through 9 December 2025, with a brief interruption from 5–10 November 2025. Dosimetric reassessment during the interruption indicated that no correction to the prescribed regimen was required, and therapy was subsequently completed as planned.

Treatment parameters, including the selected fractionation schedule (320 cGy × 17), were chosen to provide an effective therapeutic dose while balancing tolerability, treatment logistics, and the patient’s advanced age and clinical circumstances. Energy selection (70 kV) was guided by the known propensity of EMPD for adnexal extension beyond the superficial epidermis, with the aim of improving treatment coverage of potential subclinical extension [3,7].

**Figure 2:**
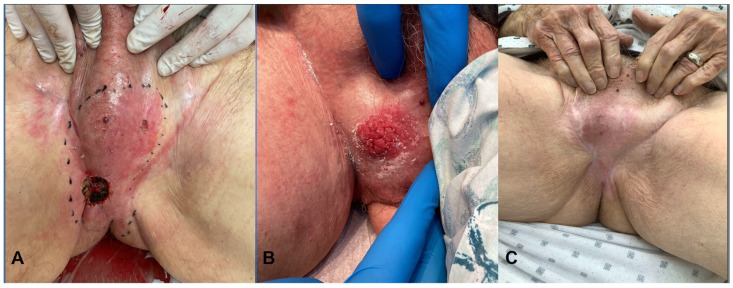
Clinical course of scrotal extramammary Paget’s disease during and after radiation therapy. (**A**) Pre-treatment presentation demonstrating an erythematous, indurated plaque with focal ulceration involving the scrotum and perineal region. (**B**) During radiation therapy, showing a friable, erythematous, and erosive lesion consistent with treatment-related changes and tumor breakdown. (**C**) Post-treatment follow-up demonstrating interval flattening with residual erythema and post-radiation skin changes, consistent with clinical response.

The patient tolerated treatment well without significant acute complications. At short-term follow-up, early clinical improvement was observed, with interval flattening of the lesion and residual post-radiation erythema. No additional treatment had been initiated at the time of last follow-up (Figure 2).

From the patient’s perspective, he expressed concern regarding the morbidity, recovery time, and potential functional impact of surgical intervention in the scrotal region, which contributed to his preference for a non-surgical treatment approach. At short-term follow-up, the patient reported tolerating treatment well and remained aligned with the decision to pursue a non-surgical approach (Table 1).

## 3. Discussion

EMPD is traditionally described as an intraepidermal malignancy; however, numerous studies have demonstrated extension along adnexal structures, including hair follicles and eccrine ducts [3,7]. This pattern of spread has direct therapeutic implications, as treatment modalities limited to superficial epidermal penetration may fail to adequately address subclinical disease. In this context, depth-informed treatment planning becomes critical when selecting non-surgical modalities, such as superficial radiation therapy. Failure to account for adnexal extension may contribute to recurrence when using superficially limited therapies, underscoring the importance of appropriate energy selection in radiation-based management.

This pattern of subclinical extension may be conceptually similar to field cancerization seen in other cutaneous malignancies, where clinically normal-appearing tissue harbors microscopic disease [3,7]. In such settings, therapies that account for both visible and subclinical involvement may be particularly important for achieving durable disease control.

The histopathologic findings were characteristic of extramammary Paget disease, with pagetoid intraepidermal proliferation and adnexal involvement [2,7]. Immunohistochemistry supported a primary cutaneous origin, with CK7 and CAM5.2 positivity [2,5]. Clinical and radiographic evaluation did not suggest an associated underlying gastrointestinal or urothelial malignancy. In the appropriate clinical context, this profile suggests that extensive malignancy workup may be of relatively low yield.

Surgical excision remains the gold standard for management; however, recurrence rates remain substantial, particularly in anatomically complex regions or when adnexal extension is present [1,4]. In elderly patients or in anatomically sensitive regions such as the scrotum, surgical management may be associated with significant morbidity, including impaired wound healing, functional compromise, and reduced quality of life [1,2]. Non-surgical approaches, including topical imiquimod, laser therapy, and radiation therapy, have been reported with variable success, often in patients who are poor surgical candidates or decline operative management [2,8]. However, these modalities are limited by heterogeneity in treatment protocols and a lack of standardized guidelines, particularly with respect to durability of response and recurrence risk. In such cases, non-surgical modalities such as radiation therapy represent an important alternative.

Prior studies have demonstrated the use of radiation therapy for extramammary Paget disease across a wide range of clinical scenarios, but with substantial variability in treatment parameters and outcomes. In one of the largest series to date, Hata et al. reported outcomes in 41 patients with genital EMPD treated with radiation therapy using total doses ranging from 45 to 80.2 Gy (median ~60 Gy) delivered over 23–43 fractions [9]. While all patients initially achieved complete clinical response, recurrence occurred in 16 of 41 patients, including local progression in 5 cases, and higher radiation doses (>60 Gy) did not consistently prevent recurrence. Notably, tumor invasion into the dermis and regional lymph node involvement were identified as significant prognostic factors, and the authors concluded that optimal radiation dosing remains undefined [9]. These findings highlight both the efficacy of radiation therapy and the persistent challenge of achieving durable local control despite dose escalation.

Similarly, smaller case series have reported favorable responses to radiation therapy but reinforce the lack of standardized treatment approaches. One study described three cases of vulvar EMPD treated with radiation doses ranging from 54 to 78 Gy, all of which achieved complete clinical response; however, one patient developed marginal recurrence despite treatment [8]. Radiation fields in this series were designed with 2–3 cm margins beyond visible disease, emphasizing a margin-based approach to treatment planning rather than biologic considerations of tumor spread [8]. In contrast, more recent reports describe successful treatment of invasive scrotal EMPD using a lower total dose of 50.4 Gy delivered in conventional fractions, with complete clinical resolution and no recurrence at short-term (6-month) follow-up [10]. However, the relatively limited follow-up duration in these reports makes it difficult to assess long-term disease control, particularly given the known risk of delayed recurrence in EMPD [10].

Despite these findings, the persistence of recurrence despite apparently adequate dosing suggests that factors beyond total radiation dose contribute to treatment failure. In the cohort reported by Hata et al., local progression occurred within the radiation field in several patients despite treatment with doses exceeding 60 Gy, indicating that dose escalation alone may be insufficient to achieve durable local control [9]. This observation raises the possibility that current treatment approaches may not fully account for the biologic behavior of EMPD, particularly its propensity for extension beyond the superficial epidermis.

In many prior studies, treatment planning has emphasized lateral field expansion using predefined margins, often in the range of 2–3 cm beyond clinically visible disease. However, as demonstrated in smaller series such as that of Son et al., marginal recurrence may still occur despite these expanded treatment fields, highlighting the limitations of margin-based approaches that primarily address horizontal spread [8]. Importantly, vertical extension along adnexal structures represents a distinct pathway of subclinical disease that may not be adequately targeted by conventional planning strategies focused on surface coverage.

Additionally, interpretation of treatment outcomes across studies is complicated by substantial heterogeneity in radiation protocols, including wide variation in total dose, fractionation schedules, and modality selection [8,9,10]. This variability makes it difficult to isolate the factors most critical for treatment success. For example, while Lin et al. reported favorable short-term control following radiotherapy for invasive EMPD, follow-up was limited to six months despite documented dermal invasion exceeding 4 mm, a depth that may have implications for long-term disease persistence [10]. Collectively, these observations suggest that inconsistent outcomes in prior reports may reflect not a failure of radiation therapy itself, but rather limitations in how treatment parameters are selected in relation to tumor biology.

In comparison to these prior studies, the present case demonstrates a more targeted, mechanism-based approach to radiation therapy planning. While total dose (5440 cGy) and fractionation were within ranges reported in the literature, treatment in this case was distinguished by deliberate selection of 70 kV energy to account for the known propensity of EMPD to extend along adnexal structures beyond the superficial epidermis [7]. Unlike prior reports, which primarily emphasize total dose and field margins, this approach integrates histopathologic understanding of tumor spread into energy selection, with the goal of improving coverage of subclinical disease [8,9,10]. Given the variability in outcomes observed across prior studies despite similar or higher radiation doses, this depth-informed strategy may represent a meaningful refinement in the application of superficial radiation therapy for EMPD, particularly in anatomically sensitive regions where surgical morbidity is a concern.

SRT offers targeted delivery of radiation, making it particularly suited for cutaneous malignancies [1]. SRT utilizes low-energy X-rays, with tissue penetration determined by kilovoltage selection, allowing for controlled deposition of dose within the epidermis and dermis [1]. Lower-energy superficial radiation beams are primarily confined to more superficial tissue layers, whereas higher kilovoltage settings provide comparatively greater dermal penetration, which may be advantageous when targeting adnexal extension in tumors such as extramammary Paget disease [1]. This distinction is particularly relevant in EMPD, where tumor cells may extend beyond the epidermis along hair follicles and eccrine ducts [3,7]. In this context, energy selection represents a critical yet underrecognized component of treatment planning, with potential implications for both local control and recurrence risk when subclinical disease is not adequately targeted.

Selection of 70 kV energy was intentionally guided by the known propensity of EMPD for adnexal extension beyond the superficial epidermis, with the aim of improving treatment coverage of potential subclinical extension [3,7]. This depth-informed approach represents a practical framework for optimizing SRT in EMPD. In this case, the selected treatment parameters were associated with early clinical improvement at short-term follow-up, supporting the potential utility of depth-guided SRT as a non-surgical management approach, although longer follow-up is required to assess durability of response and long-term disease control [3].

This case underscores the importance of tailoring dermatologic treatment of cutaneous malignancies to both tumor-specific features and individual patient factors, including age, comorbidities, anatomic location, and patient preferences. In this setting, superficial radiation therapy (SRT) may serve as a valuable addition to the dermatologic treatment armamentarium, particularly for patients who are not optimal surgical candidates.

A practical challenge encountered was the relative absence of consensus protocols for superficial radiation therapy parameters in EMPD, particularly with respect to energy selection, fractionation schedules, and total treatment duration. While surgical management remains well characterized, published data on SRT for EMPD are limited and heterogeneous, with no widely accepted protocols to guide clinical decision-making [1,8].

In this case, an individualized, multidisciplinary approach was required, with treatment parameters informed by available literature, tumor characteristics, and anatomic considerations. This highlights a broader gap in the literature and underscores the need for more standardized treatment frameworks to support clinicians utilizing radiation-based approaches for EMPD. From a clinical perspective, consideration of adnexal involvement and subclinical disease extension should play a central role in treatment selection for EMPD, particularly when utilizing non-surgical modalities [7].

Given the risk of delayed recurrence in extramammary Paget disease, close clinical surveillance remains an important component of post-treatment management, particularly following non-surgical approaches [1,4]. Although optimal follow-up intervals are not well defined, periodic clinical examination with a low threshold for biopsy of suspicious or persistent areas may be warranted. In select cases, adjunctive imaging or multidisciplinary evaluation may be considered to assess for deeper or recurrent disease. Careful longitudinal follow-up will be critical for accurately assessing treatment response and identifying late recurrence.

This report is subject to several limitations. As a single case, the findings may not be generalizable to all patients with extramammary Paget disease, particularly given the known heterogeneity in tumor biology, depth of invasion, and anatomic location. In addition, follow-up duration in this case is limited, precluding assessment of long-term disease control and the potential for delayed recurrence, which is well documented in EMPD. Histopathologic confirmation of complete tumor clearance following treatment was not obtained, and clinical response alone may not fully capture microscopic residual disease. An additional limitation is the absence of mapping biopsy, which may have provided more precise delineation of microscopic disease extent given the known potential for subclinical spread in extramammary Paget disease. Furthermore, although treatment parameters were selected based on available literature and biologic considerations, the absence of standardized superficial radiation therapy protocols limits the ability to directly compare outcomes or define optimal dosing and energy selection strategies. Future studies with larger cohorts and longer follow-up are needed to better characterize the role of depth-guided radiation therapy in EMPD management.

## 4. Conclusions

Superficial radiation therapy represents a viable non-surgical treatment option for extramammary Paget disease in carefully selected patients, particularly those with advanced age, comorbidities, or lesions in anatomically sensitive regions where surgical morbidity is a concern. In this case, selection of 70 kV energy was guided by the known propensity for adnexal extension, with the aim of improving treatment coverage of potential subclinical extension, and was associated with early clinical improvement at short-term follow-up. Although early clinical improvement was observed in this case, longer follow-up is required to assess durability of response and recurrence risk. These findings highlight the importance of incorporating tumor depth and subclinical spread into treatment planning when considering radiation-based management approaches.

## Figures and Tables

**Table 1:** Chronological summary of clinical presentation, diagnostic evaluation, treatment course, and short-term follow-up in a patient with scrotal extramammary Paget disease.

Timeline	Clinical Event
Initial presentation	88-year-old male presented with a chronic scrotal and perineal lesion
Diagnostic evaluation	Biopsy confirmed extramammary Paget disease with CK7 and CAM5.2 positivity
Staging workup	PET-CT demonstrated no evidence of metabolically active metastatic or associated visceral malignancy
Treatment decision	Surgical management discussed; patient declined due to concerns regarding morbidity and functional impact; SRT selected
7 October 2025	Superficial radiation therapy initiated
5–10 November 2025	Brief treatment interruption
9 December 2025	Treatment completed
Short-term follow-up	Early clinical improvement observed; no additional treatment initiated

## Data Availability

The original contributions presented in this study are included in the article. Further inquiries can be directed to the corresponding author.
